# Discriminating languages in bilingual contexts: the impact of orthographic markedness

**DOI:** 10.3389/fpsyg.2014.00424

**Published:** 2014-05-13

**Authors:** Aina Casaponsa, Manuel Carreiras, Jon A. Duñabeitia

**Affiliations:** ^1^Basque Center on Cognition, Brain and LanguageDonostia, Spain; ^2^Ikerbasque, Basque Foundation for ScienceBilbao, Spain; ^3^University of the Basque Country EHU/UPVBilbao, Spain

**Keywords:** bilingual reading, visual word recognition, orthographic cues, bigrams, selective lexical access

## Abstract

Does language-specific orthography help language detection and lexical access in naturalistic bilingual contexts? This study investigates how L2 orthotactic properties influence bilingual language detection in bilingual societies and the extent to which it modulates lexical access and single word processing. Language specificity of naturalistically learnt L2 words was manipulated by including bigram combinations that could be either L2 language-specific or common in the two languages known by bilinguals. A group of balanced bilinguals and a group of highly proficient but unbalanced bilinguals who grew up in a bilingual society were tested, together with a group of monolinguals (for control purposes). All the participants completed a speeded language detection task and a progressive demasking task. Results showed that the use of the information of orthotactic rules across languages depends on the task demands at hand, and on participants' proficiency in the second language. The influence of language orthotactic rules during language detection, lexical access and word identification are discussed according to the most prominent models of bilingual word recognition.

## Introduction

Bilingual societies in which two official languages coexist (e.g., Basque Country, Catalonia, Wales) have attracted a great deal of scientific attention in recent decades, given that balanced simultaneous bilinguals who are exposed to two languages on a daily basis can provide evidence about the organization of bilingual lexical representations and the mechanisms leading to effective language selection in naturalistic contexts (e.g., Costa et al., 2006; Perea et al., 2008; Duñabeitia et al., 2010b; Kuipers and Thierry, 2010). So far the evidence regarding the differences and similarities between the mechanisms guiding lexico-semantic and syntactic processing in non-simultaneous bilinguals who learn the L2 in naturalistic vs. classroom contexts is still mixed (Muñoz, 2008; Pliatsikas and Marinis, 2013, for reviews). However, results from studies testing early simultaneous balanced bilinguals living in bilingual contexts offer converging evidence on the effectiveness of cross-language activation at multiple levels of processing, especially in the visual word recognition domain (e.g., Thierry and Wu, 2007; Duñabeitia et al., 2010a). Although there is evidence demonstrating that the recognition of a visually presented word is governed by parallel access to both languages used by balanced simultaneous bilinguals, the mechanisms by which a given word form is associated with a given language (i.e., language tagging) by these bilinguals is still unclear.

In most bilingual environments readers can find different cues that help bilingual language recognition and lexical access. One extreme example of this reality is the case of languages that do not share the same script (e.g., Hebrew-English), since the individual letters that constitute the printed words are the clearest language cue. However, this situation does not apply to multiple bilingual societies in which both languages are highly similar and share the same orthography (e.g., French-English, Spanish-Basque), therefore making it difficult for readers to determine the language of each individual word. Hence, studying bilingual visual word recognition with same-script language combinations may help us to identify which are the features of the words that aid bilingual language selection and recognition.

Different languages have different orthotactic rules, and it seems plausible to assume that bilinguals could rely in such cues as a strategy while reading in an ambiguous language context. In fact, previous studies have suggested that the frequency of the letters and their combinations within a language may play an important role in bilingual language detection and to some extent may also mediate the lexical access process (Grainger and Beauvillain, 1987; Thomas and Allport, 2000; Vaid and Frenck-Mestre, 2002; Lemhöfer et al., 2008, 2011; Van Kesteren et al., 2012). Vaid and Frenck-Mestre (2002) presented English and French words to highly proficient English-French bilinguals in a speeded language decision task, and found that words that were clearly marked as belonging to one of the languages in terms of bigram frequencies (e.g., OEUF as a French-marked word) were responded to faster than unmarked words (words that follow the same orthotactic rules in both languages). These results suggest that language decision or detection could be mediated by the extraction of statistical orthographic regularities at early stages of single word processing.

In a similar vein, a recent study by Lemhöfer et al. (2011) testing compound words in a lexical decision task with monolinguals and bilinguals showed clear-cut orthographic markedness effects. They presented native and non-native Dutch participants with Dutch compound words that could contain an orthotactic parsing cue (i.e., the bigram at the morphemic boundary being a bigram that cannot exist within a Dutch morpheme), and found that the presence of such parsing cue aided morphological decomposition. In line with the results presented by Vaid and Frenck-Mestre (2002), Lemhöfer et al. concluded that the sub-lexical information at the constituent boundary might guide the identification of the individual constituents, thus helping word recognition.

Recently, Van Kesteren et al. (2012) demonstrated a language decision advantage for words that contain language-specific orthography in one of the bilingual languages, proposing a direct link between sub-lexical information of words and language membership. In their study, Norwegian-English bilinguals completed a series of language decision and lexical decision tasks including marked and unmarked Norwegian and English words, and their results demonstrated a strong reliance of bilingual readers on sub-lexical orthographic properties of words, given the clear-cut markedness effects found across tasks. They concluded that language information could be accessed directly via sub-lexical information instead of via lexical representation of words, and they proposed an extension of the Bilingual Interactive Activation Plus model in order to account for these effects. These findings closely match earlier evidence demonstrating that language-specific orthography directly affects single word identification (e.g., Vaid and Frenck-Mestre, 2002; Lemhöfer et al., 2008, 2011), suggesting that access to the lexicon might be guided by the extraction of language-specific orthotactic combinatorial rules. That is, at early stages of visual word identification, language selection mechanisms have been proposed to operate enhancing lexical activation of the relevant language on the basis of the sub-lexical structure of the words (see Grainger and Beauvillain, 1987; Schwartz et al., 2007; see also Westbury and Buchanan, 2002, for evidence in monolinguals).

The main goal of the current study is to better understand bilingual language identification processes by exploring the influence exerted by the sub-lexical characteristics of the words in bilinguals' two languages during different stages of word recognition. We tested L2 words that could be either legal or illegal in the L1 vocabulary in terms of their corresponding bigram frequencies in two different tasks with varying demands of lexical access: a language decision task and a perceptual identification task. To this end, the materials included Basque-specific words (e.g., ETXE [house]; note that TX is an illegal bigram in Spanish) and orthographically unmarked words (e.g., MENDI [hill]; note that all the bigrams are also plausible in Spanish). Besides, in order to explore whether or not the reliance on L2 orthotactic cues depends on the degree of L2 proficiency, three different samples of participants were tested: balanced Spanish-Basque bilinguals, highly-proficient unbalanced bilinguals (L1 Spanish, L2 Basque) and Spanish monolinguals.

Interestingly, and contrary to any explanation of the markedness effect in terms of sub-lexical-lexical interactions, Vaid and Frenck-Mestre (2000) proposed that the markedness effect *“is predominantly a perceptual effect rather than one involving complete lexical access”* (p. 52). It should be noted that the term “perceptual” refers to an effect based on orthographic information of words rather than on perceptual features of letters. That is, they suggested that participants could have taken their language decisions following a sub-lexical strategy, without relying on the real meaning of the marked words. Rather than discriminating L1 and L2 based on the lexical representations in each language, participants could have taken a different strategy and could have completed the task by simply deciding whether or not a given string corresponds to the L1. In other words, rather than identifying OUEF as a French word by accessing its meaning, participants could have simply discarded it as an English word given its orthotactic regularities. Such an account is difficult to reject on the basis of the existing evidence. However, one possible solution to the conundrum is to include a group of monolinguals in the experiment and to compare their performance to that of bilinguals. If participants exclusively rely on low-level L1 orthographic rules to perform language discrimination tasks, even monolinguals would benefit of the presence of L2-marked words, showing facilitative effects for strings containing orthotactic cues regardless of their L2 knowledge. Put differently, if the L2 markedness effect exclusively relies on a sub-lexical strategy based on the detection of L1 orthographic violations, extensive knowledge of the L2 is not required to complete the language detection task, given the sub-lexical locus of the decision criteria. Hence, even monolinguals who are not familiar with the L2 could perform correctly on the basis of this account. At the same time an inhibitory effect is expected for monolinguals compared to bilinguals for non-marked L2 words due to the similarity with real words (see Westbury and Buchanan, 2002; Lemhöfer et al., 2008, for a review).

As previously mentioned, most of the studies exploring the L2 word markedness effect have used tasks that do not explicitly require full lexical access to the written representations, given that these studies have mainly used the language decision task or the (mixed) language lexical decision task (see Vaid and Frenck-Mestre, 2002; Van Kesteren et al., 2012). One of the main problems with these tasks is that it is difficult to estimate the degree of lexical access needed to efficiently determine whether a given string corresponds to language X or Y, or whether it is a real or invented word, given the difficulty to estimate the impact of factors associated with word likelihood (e.g., Jacobs and Grainger, 1994; Jacobs et al., 1998; see Wagenmakers et al., 2004, for review). Hence, in order to disambiguate between proposals claiming for a different influence of L2 orthotactic cues in sub-lexical ortgographic decisions, on the one hand, and in lexico-semantic access, on the other, and following the line opened by Van Kesteren et al. who suggested that the L2 markedness effect may largely depend on the specific task demands, in the present study we investigated the presence of this effect in two tasks, one of which explicitly requires conscious access to the specific visually presented representation. Participants' performance in a language decision task (Experiment 1) was compared to their performance in a perceptual identification task (Experiment 2). The perceptual identification task selected was the progressive demasking task (PDM hereafter) developed by Grainger and Segui (1990) and implemented by Dufau et al. (2008). The PDM is a perceptual task that requires participants to recognize letter strings by pressing a button key and to write them back on the keyboard (for different applications of this task, see Carreiras et al., 1997; Duñabeitia et al., 2008). Importantly, the PDM task does not allow for responses based on a mere strategy of estimating the L1-membership likelihood on the basis of specific letter combinations, since the whole string needs to be retained in memory to correctly complete the task. Given the difficulty to access and remember L2-marked strings for monolingual participants who presumably have never faced the critical L1-illegal bigrams, it seems reasonable to tentatively predict that marked words would help bilinguals' performance in this task, while the opposite pattern is expected for monolingual participants. Besides, since monolingual participants will need to complete this task by following an orthography-to-phonological working memory strategy instead of a lexical strategy, they would take longer to recognize letter strings including letter combinations that are not present in their L1 as compared to words that follow the L1 orthographic rules (i.e., L2 unmarked words). It was also expected that, overall, L2 words would be harder to recognize for unbalanced than for balanced bilinguals, given that the speed and accuracy of lexical access is highly sensitive to proficiency (see, among many others, Dimitropoulou et al., 2011; Francis et al., 2014).

## Experiment 1: speeded language decision task

### Materials and methods

#### Participants

Sixty undergraduates (44 women; mean age = 23.11, *SD* = 3.70) with normal or corrected-to-normal vision participated in this experiment in exchange for monetary compensation. Twenty were balanced Spanish-Basque bilinguals from the Basque Country (12 women; mean age = 24.54, *SD* = 5.29). These balanced bilinguals had a native-like proficiency in both Basque and Spanish, as calculated by their proficiency self-ratings (see Table 1). A group of 20 unbalanced bilinguals was also selected, being all of them native Spanish speakers from the Basque Country (14 women; mean age = 21.73, *SD* = 2.78) who learnt Basque as a second language and were relatively high proficient in Basque, but not native-like (see Table 1). The remaining 20 participants were Spanish monolinguals (18 women; mean age = 23.05, *SD* = 3.03) with no prior knowledge of Basque. The overall self-perception level of Spanish ranged from 9 to 10 for all groups of participants (mean = 9.73, *SD* = 0.45). Balanced bilinguals also ranged from 9 to 10 in their knowledge of Basque (mean = 9.62, *SD* = 0.50), and unbalanced bilinguals ranged from 6 to 8 in their self-perceived Basque proficiency (mean = 7.54, *SD* = 0.71). The monolinguals had never learnt Basque and all of them lived in Murcia, a monolingual region of Spain. None of the participants reported neurological or psychiatric disorders. All participants gave their written informed consent in accordance with guidelines approved by the Ethics and Research Committees of the Basque Center on Cognition, Brain and Language. The study was also performed in accordance with the ethical standards set in the Declaration of Helsinki.

**Table 1:** **Mean levels of Spanish and Basque language proficiency calculated according to participants' self-ratings (in a 1-to-10 scale)**.

**Language proficiency**	**Balanced**		**Unbalanced**		**Monolinguals**	
	**Spanish**	**Basque**	**Spanish**	**Basque**	**Spanish**	**Basque**
Speaking	9.85 (0.46)	9.62 (0.64)	9.88 (0.33)	7.08 (0.89)	9.75 (0.55)	–
Understanding	9.88 (0.33)	9.81 (0.40)	9.92 (0.27)	8.42 (0.86)	9.50 (0.61)	–
Writing	9.69 (0.55)	9.46 (0.81)	9.65 (0.75)	6.92 (1.26)	9.68 (0.47)	–
Reading	9.88 (0.33)	9.81 (0.49)	9.77 (0.65)	8.12 (1.14)	9.48 (0.72)	–
General self-perception	9.81 (0.40)	9.61 (0.50)	9.73 (0.45)	7.54 (0.71)	9.45 (0.76)	–

Standard deviations are provided within parentheses.

#### Stimuli

Six hundred and eighty words were used as targets. Half of them were Spanish words taken from Davis and Perea (2005) and the other half were Basque words taken from Perea et al. (2006). Critically, Basque words were selected as a function of their bigram combinations so that they could be either valid o invalid in both languages. Half of the Basque words were marked by bigram combinations that were only plausible in Basque (i.e., L2-marked words; e.g., *txakur* [dog], where the bigram “tx” do not exist in Spanish), and the other half were unmarked words that also followed the Spanish orthotactic rules [*mendi* (hill)]. Marked Basque words were always formed by at least one illegal bigram when measured according to the Spanish vocabulary. Besides, their mean bigram frequency when measured in Spanish fell below the mean log10 frequency of all existing Spanish bigrams as measured from LEXESP (Sebastián-Gallés et al., 2000). In contrast, unmarked Basque words were formed by valid bigram combinations in both languages with mean bigram frequencies falling above the mean log10 Spanish bigram frequency distribution. Spanish words were also split in two sets that were carefully matched between them. One of the Spanish set was assigned as matched control for Basque marked words and the other one was selected as a control for Basque unmarked words. All possible sub-lexical and lexical factors were equated across and within sets (see Table 2).

**Table 2:** **Mean values for each sub-lexical, lexical, and semantic factor of the L1 (Spanish) and L2 (Basque) word used split by condition**.

	**BASQUE**		**SPANISH**	
	**Marked**	**Unmarked**	**Control marked**	**Control unmarked**
Word frequency	52.00 (114.53)	47.36 (109.53)	44.65 (81.17)	42.56 (74.86)
Word length	6.62 (1.83)	6.81 (2.22)	6.81 (1.81)	6.82 (1.77)
Number of orthographic neighbors	1.42 (1.62)	1.55 (0.35)	1.53 (2.74)	1.69 (3.01)
Age of acquisition	3.22 (0.49)	3.23 (0.50)	3.19 (0.56)	3.19 (0.61)
Word concreteness	4.09 (0.89)	4.12 (0.86)	4.05 (0.81)	4.07 (0.85)
Spanish bigram frequency	1.72 (0.3)	2.97 (0.24)	2.49 (0.30)	2.46 (0.33)
Basque bigram frequency	2.88 (0.18)	2.89 (0.20)		
Number of spanish-implausible bigrams	2.35 (0.93)	0 (0)		

Standard deviations are provided within parentheses.

#### Procedure

Participants were tested individually in a quiet room using DMDX software (Forster and Forster, 2003) on a 15” monitor set at 90 Hz. Stimuli were presented in lowercase Courier New white letters on a black background. First, a fixation point appeared on the screen for 500 ms followed by the target until participants' response (or for 2500 ms). Feedback was provided only when participants made a mistake. Participants were asked to respond with the right hand to Basque words and with the left hand to Spanish words using a response box. Trial presentation order was randomized across participants. Twenty practice trials were included prior to the experimental trials. The experimental session approximately lasted for approximately 30 min.

## Results and discussion

**Figure 1:**
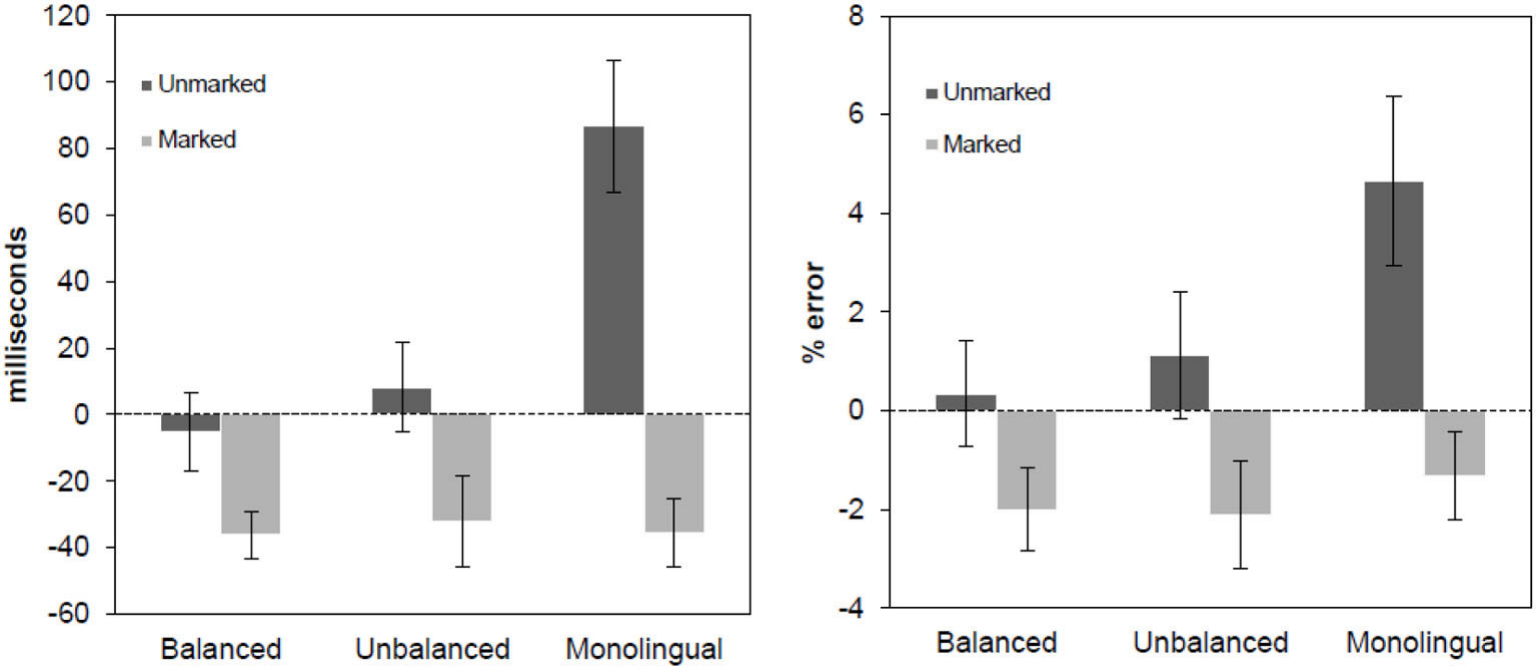
**Language effect in reaction times (left panel) and error rates (right panel) for speeded language decision task, separated by marked and unmarked conditions**. The effect was obtained subtracting the responses to the Spanish word from the responses to the Basque words. Error bars represent 95% confidence intervals.

Erroneous responses were excluded from the latency analysis as well as responses above or below 2.5 standard deviations from the participants-based and items-based means in each condition (4.80% Balanced Bilinguals, 4.60% Unbalanced Bilinguals, 4.52% Monolinguals). ANOVAs on mean latencies for correct responses and error rates were conducted following a 3 (Group: Balanced Bilinguals, Unbalanced Bilinguals, Monolinguals) × 2 (Language: Spanish, Basque) × 2 (Bigram: Marked, Unmarked) design. Comparisons of the effects were also conducted within and between groups by subtracting the RTs and error rates in Basque trials from the RTs and error rates in the Spanish trials. Mean latencies and error rates are presented in Table 3 and the effects are plotted in Figure 1.

**Table 3:** **Mean latencies (in milliseconds) and error rates (in percentage) for words in the four conditions and participant groups for speeded language decision task (Experiment 1)**.

	**Balanced**		**Unbalanced**		**Monolingual**	
	**RT**	**Error rate**	**RT**	**Error rate**	**RT**	**Error rate**
L2 unmarked	667 (75)	3.35 (2.79)	682 (113)	5.21 (3.82)	689 (128)	8.16 (5.40)
L2 marked	631 (72)	1.97 (1.60)	635 (95)	2.30 (2.24)	564 (87)	1.56 (1.59)
L1 control unmarked	672 (77)	3.01 (1.54)	674 (112)	4.09 (1.90)	603 (102)	3.50 (2.95)
L1 control marked	667 (74)	3.98 (2.14)	667 (113)	4.41 (2.25)	600 (128)	2.88 (2.34)
Unmarked effect	−5 (27)	0.35 (2.46)	8 (30)	1.12 (2.97)	87 (20)	4.66 (3.92)
Marked effect	−36 (16)	−2.00 (1.92)	−32 (31)	−2.12 (2.48)	−36 (10)	−1.32 (0.90)

Standard deviations of the means are provided within parenthesis.

### Reaction times

The main effect of Language was not significant [*F*1/*F*2 < 0.85, *p*s > 0.35]. The main effect of Bigrams was significant [*F*1_(1, 57)_ = 171.34, *p* < 0.001; *F*2_(1, 676)_ = 112.23, *p* < 0.001], suggesting that marked words were recognized faster than unmarked words. The main effect of group did not reach significance in the analysis by participants, but it was significant in the by-item analysis [*F*1_(2, 57)_ = 1.72, *p* = 0.19; *F*2_(2, 1352)_ = 202.99, *p* < 0.001]. Critically, the three-way interaction was significant [*F*1_(2, 57)_ = 36.49, *p* < 0.001; *F*2_(2, 1352)_ = 56.13, *p* < 0.001]. For Basque marked words, all groups tended to respond faster to them than to their corresponding Spanish control words (all *t*s > 4.5 and *p*s < 0.001). Furthermore, this markedness effect (i.e., Basque marked words minus Spanish control words) was similar across all groups of participants (all *t*s < 0.6, *p*s > 0.55). In contrast, a different pattern emerged for unmarked Basque words. Balanced and Unbalanced Bilinguals responded similarly to unmarked Basque words and to their Spanish controls (all *t*s < 1.5 and *p*s > 0.25), while monolinguals took more time to recognize unmarked Basque words than Spanish controls (i.e., an inhibitory effect; [*t*_(19)_ = −8.59, *p* < 0.001]) (see Table 3).

### Error rates

The statistical analysis on the accuracy data fully replicated the pattern observed in the RTs. The main effect of Language was not significant [*F*1/*F*2 < 0.2, *p*s > 0.65] and the main effect of Bigram was significant [*F*1_(1, 57)_ = 52.23, *p* < 0.001; *F*2_(1, 676)_ = 17.51, *p* < 0.001], showing more errors for unmarked than for marked words. Again, the main Group effect did not reach significance in the by-participants analysis [*F*1_(2, 57)_ = 1.84, *p* = 0.31; *F*2_(2, 1352)_ = 6.64, *p* < 0.005]. Critically, the three-way interaction was significant [*F*1_(2,57)_ = 5.65, *p* < 0.01; *F*2_(2, 1352)_ = 5.15, *p* < 0.01], showing the same pattern of results observed in the RT analysis. In general, participants made fewer errors with Basque-marked words than with Spanish control words (all *ts > 2.5*, *p* < 0.05) and the magnitude of the effects (i.e., the differences between Basque marked words and theirs Spanish control words) did not differ between groups (all *t* < 0.15, *p* > 0.25). For unmarked Basque words, the accuracy rates were similar to that for Spanish control words in the groups of Balanced and Unbalanced Bilinguals (*t*s < 2 and *p*s > 0.1). In contrast, Monolinguals made more errors on unmarked Basque words than on their Spanish controls [*t*_(19)_ = −5.32, *p* < 0.001].

In general, L2 words that violated the orthotactic rules of Spanish vocabulary (i.e., marked Basque words) were easier to recognize than Spanish words for all groups (faster RTs and lower error rates), suggesting that readers base their decisions regarding the language membership of the words based on orthographic cues. Interestingly, this was true for the two groups of bilinguals, regardless of their clear-cut differences in Basque proficiency, and more strikingly, this was also true for the group of monolinguals with no prior experience with Basque. This result raises a critical question regarding the etiology of this effect. The fact that all participants showed identical markedness effects for L2-specific Basque words suggests that the cognitive processes underlying language discrimination of orthographically-marked words are guided by basic sub-lexical processes associated with the detection of non-native bigram combinations (i.e., the detection of L1-invalid cues; see Vaid and Frenck-Mestre, 2002).

Another critical finding from Experiment 1 helps us qualifying the real cognitive mechanisms leading to efficient language discrimination in bilinguals and monolinguals. Basque words following the Spanish orthotactic rules (i.e., unmarked Basque words) were notably difficult to recognize for monolinguals, but not for bilinguals. This effect of unmarked Basque words that was only present for monolinguals, together with the results observed for marked Basque words across the three groups of participants, suggest that there are two clearly different mechanisms driving language detection depending on the specific orthographic characteristics of the words. First, some form of lexical access seems to determine language detection mechanisms for unmarked words, given the obvious differences in the performance of bilinguals and monolinguals with these stimuli (namely, an inhibitory effect only present in the group of monolinguals, who lack a lexical representation for those items). Second, decisions to L2-marked words seem to be governed by a series of visuo-orthographic processes, rather than by lexical access, given the highly similar performance of all groups with marked Basque words.

In order to better characterize the importance of orthographic cues in bilingual lexical access, and to explore in depth the extent to which visuo-orthographic and lexico-semantic mechanisms determine bilingual visual word recognition we run a second experiment. In Experiment 2 we asked the same groups of participants to perceptually recognize the same Spanish and Basque (marked and unmarked) words in a progressive demasking task. Since correctly completing this task requires retaining the whole strings of letters in memory, a lexically-mediated recognition strategy would yield higher efficiency (shorter reaction times and lower error rates) during the task. Letter strings that have an actual lexical node would be encoded in episodic memory for posterior retrieval more efficiently than letter strings that are not represented in the lexicon. Therefore, we expected that bilinguals would benefit from the presence of such an entry in the lexicon compared to monolinguals. Furthermore, we expected lower activation thresholds of L2 lexical items for balanced bilinguals compared to unbalanced bilinguals reflected in shorter reaction times. Considering the characteristics of the task used in Experiment 2, we predicted that Basque words should take longer to recognize (and lead to higher error rates) than Spanish words for monolinguals and unbalanced bilinguals, but not for balanced bilinguals (who share two L1s). Given the importance of orthographic cues for all groups of participants (as seen in Experiment 1), we predicted that for balanced and unbalanced bilinguals marked Basque words should be recognized faster than unmarked words. In contrast, monolinguals should display now either similar or more difficulty in recognizing words containing letter combinations that are not present in their language, given that the encoding in working memory of letter sequences that have not been faced beforehand would require a costly perceptual and orthographic analysis of each stimulus.

## Experiment 2: progressive demasking task (PDM)

### Participants and stimuli

These were the same as in Experiment 1.

### Procedure

Participants were asked to identify the displayed words as fast and as accurately as possible typing on the keyboard the word they think they read. The experiment was run using the PDM software (Dufau et al., 2008). Trials were composed of target–mask pairs that were consecutively repeated several times. In each trial, the total display time of the stimulus was held constant at 210 ms, and the ratio of the target and mask display durations progressively increased in cycles. In the first cycle, the mask display duration was much longer than the target one (195 and 15 ms, respectively). In the following cycles, the mask display duration decreased and the target display duration increased in a constant way. Participants had to press the spacebar when they had recognized the word, and then type it. Reaction times (RTs) were measured from the initial display of the mask in the first cycle to the button press.

## Data analysis

**Figure 2:**
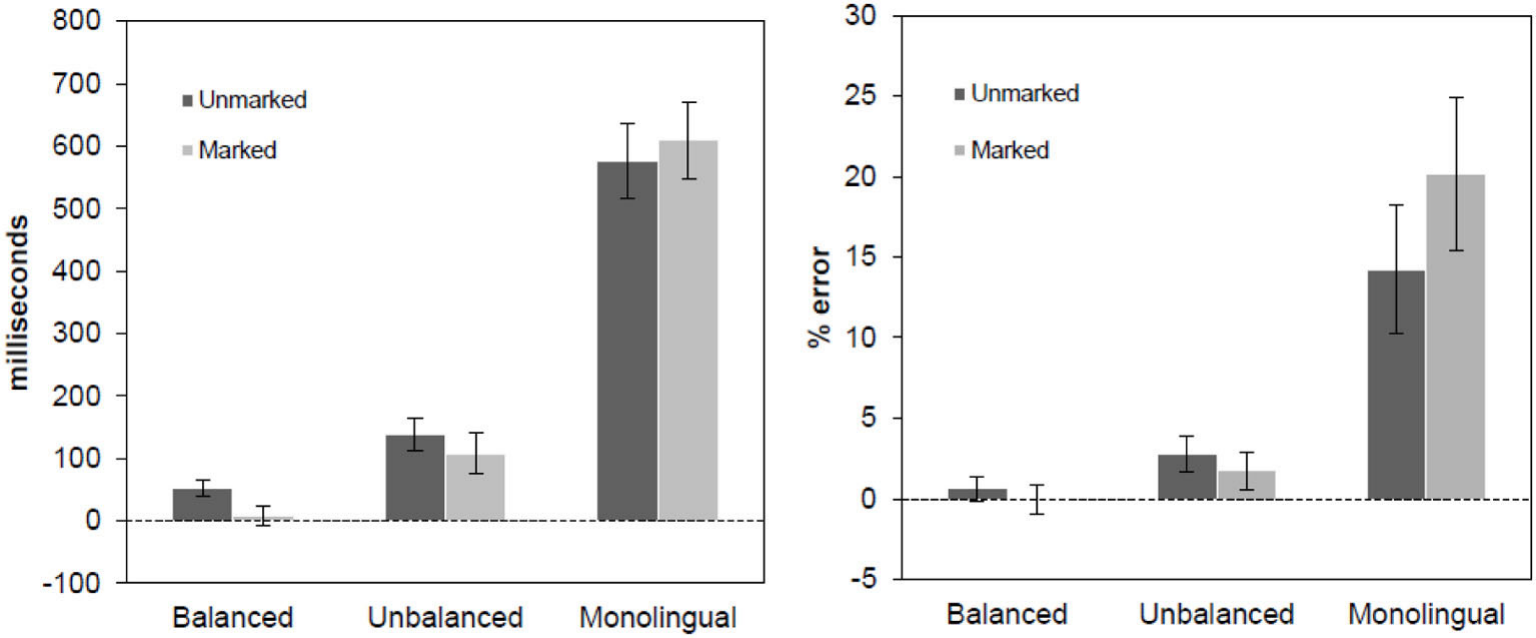
**Language effect in reaction times (left panel) and error rates (right panel) for PDM task, separated by markedness conditions**. The effect was obtained subtracting the responses to the Spanish word from the responses to the Basque words. Error bars represent 95% confidence intervals.

Erroneous responses and responses above and below 2.5 standard deviations from the mean of each subject within each condition and of each item were excluded from the analysis of reaction times (2.93% for Balanced Bilinguals, 3.48% for Unbalanced Bilinguals and 3.98% for Monolinguals). The same design from Experiment 1 was followed for the ANOVAs. Mean latencies and error rates are presented in Table 4 and effects are plotted in Figure 2 (upper panel).

**Table 4:** **Mean latencies (in milliseconds) and error rates (in percentage) for words in the four conditions and participant groups for progressive demasking task (Experiment 2)**.

	**Balanced**		**Unbalanced**		**Monolingual**	
	**RT**	**Error rate**	**RT**	**Error rate**	**RT**	**Error rate**
L2 unmarked	1404 (234)	2.65 (2.19)	1438 (225)	4.71 (2.62)	2064 (305)	16.88 (10.42)
L2 marked	1351 (227)	2.68 (2.44)	1481 (228)	4.21 (2.79)	2067 (306)	22.88 (11.77)
L1 control unmarked	1352 (223)	2.03 (1.80)	1343 (214)	1.91 (1.69)	1487 (236)	2.65 (1.96)
L1 control marked	1343 (216)	2.74 (1.90)	1330 (191)	2.47 (1.44)	1458 (224)	2.71 (1.61)
Unmarked effect	52 (30)	0.62 (1.60)	138 (26)	2.79 (2.55)	557 (137)	14.24 (3.03)
Marked effect	8 (35)	−0.05 (2.04)	108 (75)	1.74 (2.69)	609 (140)	20.18 (10.89)

Standard deviations of the means are provided within parenthesis.

## Results and discussion

### Reaction times

The main effect of Language was significant [*F*1_(1, 57)_ = 481.24, *p* < 0.001; *F*2_(1, 676)_ = 290.20, *p* < 0.001], showing that Spanish words were recognized faster than Basque words. The main effect of Bigram was also found in the analysis by participants [*F*1_(1,57)_ = 55.59, *p* < 0.001; *F*2_(1,676)_ = 1.69, *p* = 0.21]. The main effect of Group was significant [*F*1_(2,57)_ = 18.59, *p* < 0.001; *F*2_(2,1352)_ = 1982.76, *p* < 0.001], suggesting that monolinguals in general were slower recognizing words than bilinguals. The three-way interaction was significant [*F*1_(2,57)_ = 14.18, *p* < 0.001; *F*2_(2,1352)_ = 5.55, *p* < 0.05]. All groups responded significantly slower to unmarked Basque words than to the Spanish control words (all *t*s > 7.5, *p*s < 0.001), while a different pattern emerged for marked Basque words. While Unbalanced bilinguals and Monolinguals were significantly slower in responding to marked Basque words than to the corresponding Spanish control words (all *t*s > 6.4 and *p*s < 0.001), no such difference was found for Balanced bilinguals, who recognized marked Basque words as fast as the Spanish control words [*t*_(19)_ = −1.04, *p* > 0.3]

The differences in the magnitude of the effects between unmarked Basque words and their Spanish control words were also different across all three groups (all *t*s > 5.5, *p*s < 0.001), increasing as an inverse function of their proficiency in the language (see Figure 2). In contrast, the markedness effect also increased as an inverse function of the participants' proficiency in Basque (all *t*s > 5.35 and *p*s < 0.001). Interestingly, the analysis of the magnitude of the effects between marked and unmarked words revealed a facilitative effect for the two bilingual groups [Balanced bilinguals: *t*_(19)_ = 4.96, *p* < 0.001; Unbalanced bilingual: *t*_(19)_ = 3.21, *p* < 0.005], showing that L2-marked words were recognized faster than L2-unmarked words, and an inhibitory effect for the monolingual group [*t*_(19)_ = −2.44, *p* < 0.05], showing that L2-marked words were more difficult to recognize than L2-unmarked words.

### Error rates

The main effect of Language was significant [*F*1_(1, 57)_ = 78.16, *p* < 0.001; *F*2_(1, 676)_ = 199.53, *p* < 0.001], showing that in general Spanish words were recognized more accurately than Basque words. The main effect of Bigram was also significant [*F*1_(1, 57)_ = 22.58, *p* < 0.001; *F*2_(1, 676)_ = 3.01, *p* < 0.05], as well as the main effect of Group [*F*1_(2, 57)_ = 33.16, *p* < 0.001; *F*2_(2, 1352)_ = 229.05, *p* < 0.001], showing that monolinguals made more errors than both bilingual groups. Critically, the three-way interaction was significant [*F*1_(2, 57)_ = 21.20, *p* < 0.001; *F*2_(2, 1352)_ = 9.46, *p* < 0.001], showing a different pattern of the effects for the three types of participants. Not surprisingly, Balanced bilinguals did not show any reliable difference across all conditions (all *t*s < 1.75 and *p*s > 0.1), given their high and comparable degree of proficiency in the two languages. In contrast, Unbalanced bilinguals and Monolinguals responded more accurately to Spanish words than to Basque marked and unmarked words (all *t*s > 2.85 and *p*s < 0.01), and the error rates decreased as a function of increased proficiency in Basque (all *t*s > 2.37 and *p*s < 0.05). Interestingly, the magnitude of the effects between marked and unmarked words revealed an inhibitory effect for the monolingual group [*t*_(19)_ = −5.06, *p* < 0.001], showing that Spanish monolingual participants made more errors typing L2-marked words than L2-unmarked words. No statistical differences were found for any of the bilingual groups [all *t*s < 1.5, *p*s > 0.15].

The results of Experiment 2 were clear-cut. Balanced bilinguals took the same amount of time to identify Spanish words and marked Basque words, while in all the other groups and conditions, a generalized identification cost was evident for Basque (L2) words. Also, participants made more errors when typing L2 words than L1 words, but again balanced bilinguals did not show any difference in their accuracy of response to Spanish and Basque words. Interestingly, different markedness patterns emerged for monolinguals as compared to both balanced and unbalanced bilinguals. Monolinguals took more time and made more errors in recognizing L2-marked than unmarked words, but the opposite pattern was found for both types of bilinguals who showed faster responses for L2-marked than unmarked words and no significant differences in terms of accuracy. Thus, these results suggest that different mechanisms or strategies are involved in the recognition of strings of letters that include legal and illegal bigram combinations, depending on the existence of lexical representations associated with the target strings (i.e., a lexical strategy vs. an orthographic-to-phonological working memory strategy).

Together, these results suggest that (1) bilingual participants followed a lexical-search strategy when recognizing marked and unmarked words, while monolinguals followed a different encoding strategy, and that (2) L2-marked words help bilingual lexical access, leading to advantageous word identification as compared to words that orthographically speaking can also belong to their L1. This suggests that bilinguals rely on both sub-lexical and lexical information during multilingual perceptual identification of words.

## General discussion

The main goal of the present study was to investigate how the sub-lexical characteristics of the words from bilinguals' two languages influence different stages of the visual word recognition process. Three groups of participants (balanced bilinguals, unbalanced bilinguals, monolinguals) were tested in a language decision task and in a progressive demasking task. Materials consisted of a selection of Spanish (L1) and Basque (L2) words. Crucially, L2 words could follow L1 orthotactic rules (i.e., language-unspecific orthography; L2-unmarked words) or violate L1 orthotactic rules in terms of the corresponding bigram frequencies (i.e., language-specific orthography; L2-marked words). Results showed that L2-marked and unmarked words were recognized differently depending on the task demands and on the participants' linguistic profile. When the task required explicitly focus on the language tag (Experiment 1), all group of participants showed strong markedness effects (namely, an advantage in the recognition of L2-marked words) independently of their L2 knowledge and proficiency. Language-specific orthography speeded up participants' language decisions. However, when the task required participants to fully identify the strings (Experiment 2), the L2-markedness advantage only emerge for bilingual participants. Additionally, these effects were clearly modulated as a function of bilinguals' L2 lexical knowledge and proficiency.

As seen in Experiment 1, all participants seem to have based their language decisions on the existing sub-lexical cues, as reflected by the generalized benefit for L2-marked words (which were recognized even faster than L1 words). Critically, this effect was present for all types of participants, regardless of their knowledge of the L2 and their proficiency in that language. At first glance, this result could be taken as evidence supporting the sub-lexical strategy that has been suggested to guide bilinguals' language identification (see Vaid and Frenck-Mestre, 2002). Furthermore, results from Experiment 1 could be taken as a confirmation of the existence of tight links between sub-lexical information and language membership (see Van Kesteren et al., 2012). However, a closer look at the effects found in Experiment 1 for L2-unmarked words suggests that the sub-lexical strategy is not the only mechanism at play during language discrimination. When such orthotactic cues were not available to participants (namely, L2-unmarked words), all participants (bilinguals and monolinguals) seem to have followed a lexical-search strategy, given that they did not have any cue other than the match between the printed string and their known lexical forms to assign the language. Bilinguals performed notably well with L2-unmarked words, given the existence of L2 lexical representations associated with these strings, while this was not the case for monolinguals. Monolinguals took more time to recognize L2-unmarked words, most probably due to an intensive and fruitless lexical search for those items.

These two different strategies (lexical vs. sub-lexical) are correctly accommodated by current models of bilingualism (i.e., BIA+, Dijkstra and Van Heuven, 2002; and the extension of the BIA+, Van Kesteren et al., 2012), insofar they suggest that language membership can be accessed (1) once the lexical representations are activated, and also (2) directly from sub-lexical levels of processing. Our results help to better defining the specific situations in which these two routes are followed, clarifying the specific scenarios in which the sub-lexical strategy may be useful. On one hand, all readers seem to follow the sub-lexical strategy for L2-marked words. Hence, bilinguals seem to base their judgments for L2-marked words on a sub-lexical strategy that is also shared by monolinguals. On the other hand, when no orthographic cues are available (L2-unmarked words) participants follow a lexical strategy based on the identification of the correspondent word form in the lexicon (and hence the differences between monolinguals and bilinguals). However, according to this lexical search strategy, a clear modulation of the effects for L2-unmarked words would have also been expected within the two bilingual samples, given their obvious L2-proficiency differences. Nonetheless, this proficiency effect for L2-unmarked words was absent in Experiment 1, since the effect was highly similar for both groups of bilinguals. We tentatively proposed that the language decision task might not be sensitive enough to capture these subtle differences based on participants' L2 proficiency, and Experiment 2 confirmed this intuition.

Experiment 2 qualified, complemented and extended the observations from Experiment 1. First, the results from the progressive demasking task suggest that when language membership assignment is not the main aim of the task, participants mainly rely on their lexicon, partially abandoning the sub-lexical strategy followed in Experiment 1 and focusing on their lexical knowledge. Balanced bilinguals performed similarly with L1 and L2 words, while unbalanced bilinguals were significantly slower and made more errors for L2 words than for L1 words. Besides, monolinguals were markedly slow and inaccurate in identifying Basque (unknown) words. Hence, in contrast to Experiment 1, Experiment 2 clearly showed a graded pattern of effects associated with proficiency.

Critically, all bilinguals (balanced and unbalanced) showed a benefit in their speed of recognition for L2-marked words as compared to L2-unmarked words, suggesting that even in a task in which language membership assignment is not required, early detection of the language through a sub-lexical analysis of the words aids lexical access. On the basis of their statistical regularities, L2-unmarked words would initially activate Spanish and Basque lexical candidates, while L2-marked words would provide bilingual readers with a critical cue exclusively pointing to the Basque vocabulary (see Grainger and Beauvillain, 1987; Schwartz et al., 2007). Obviously, these cues would not be helpful for monolinguals, given the absence of a Basque lexicon. These results fit well with the postulates of the extension of the BIA+ model proposed by Van Kesteren et al. (2012), who suggested that information regarding language-specific sub-lexical information aid language detection. Importantly the present results extend their claims by showing that even in a context in which assignment of language membership is not required, sub-lexical cues aid lexical search by inhibiting lexical representations from the non-target language or aiding the selection of the target language, thus facilitating lexical access. In the absence of these orthographic cues, the multiplicity of activated lexical candidates from the L1 and the L2 results in a high degree of dispersion of the activation, leading to an enhanced difficulty in selecting the correct representation from the lexicon. These results fit well with some of the mechanisms proposed in the BIA+ model (Dijkstra and Van Heuven, 2002), demonstrating that when a printed word is presented to a bilingual, both languages would be initially activated (non-selective access), but as proposed by Van Kesteren et al., in the presence of orthographic cues the sub-lexical features would allow for certain degree of selective lexical access. Moreover, our results also fit well with the dual-route account specified in the BIA+ extended model. According to Van Kesteren et al., language membership information could be accessed through the retrieval of the lexical information of the words, or directly via sub-lexical information of the letter strings. When the goal of the task is to detect language membership (e.g., language decision tasks; Experiment 1), task-related decisions could be made based on the direct links established between sub-lexical nodes and language membership, making full lexical access unnecessary. That is, L2-marked words could be detected just following a sub-lexical strategy. However, when lexical access is required to correctly perform the task (e.g., word identification tasks; Experiment 2), the sub-lexical route remains effective, but decisions are also mediated by a lexical search strategy. That is, L2-marked words in a word identification task would simultaneously activate both the lexical and sub-lexical routes, which are interconnected following interactive activation principles, thus facilitating bilingual single word recognition.

It is well known that sub-lexical orthographic regularities of the words have a direct impact in the way in which monolingual readers decipher the written code, as shown by multiple studies demonstrating the impact of bigram frequencies in visual word recognition (e.g., Whitney, 2001; Grainger and Van Heuven, 2003; Whitney and Cornelissen, 2008; Dandurand et al., 2011). However, to date little is known about the impact of these orthographic regularities in bilingual reading, and moreover, about the manner in which these regularities can be unconsciously used as access cues to the bilingual lexicon. The results reported in this article demonstrate the importance of sub-lexical orthographic features in bilingual reading by showing a high degree of sensitivity of bilingual readers to language-specific bigram combinations that is strikingly different from the pattern seen in monolingual readers under the appropriated experimental contexts. In summary, we have shown that L2-marked words are always faster to recognize than L2-unmarked words for individuals who are immersed in bilingual contexts (but not for monolinguals), independently of the task demands. Besides, we have shown that the reliance on sub-lexical information seems to depend on the specific nature of the task and, more importantly, on the proficiency of the participants in the second language, in spite of their permanent exposure to the two languages in a naturalistic context. The current results demonstrate the existence of (at least) two possibly interconnected strategies during bilingual lexical access: a sub-lexical visuo-orthographic stage that is highly sensitive to the specific language cues, and a lexical search strategy. Thus, the differences between the orthotactic rules of two languages that share the same script are extremely important for language detection, and ultimately for lexical access in bilingual contexts.

### Conflict of interest statement

The authors declare that the research was conducted in the absence of any commercial or financial relationships that could be construed as a potential conflict of interest.
